# Impacts of Dynamic LED Lighting on the Well-Being and Experience of Office Occupants

**DOI:** 10.3390/ijerph17197217

**Published:** 2020-10-02

**Authors:** Rongpeng Zhang, Carolina Campanella, Sara Aristizabal, Anja Jamrozik, Jie Zhao, Paige Porter, Shaun Ly, Brent A. Bauer

**Affiliations:** 1 Well Living Lab, Rochester, MN 55902, USA; carolina.campanella@delos.com (C.C.); sara.aristizabal@delos.com (S.A.); a.jamrozik@gmail.com (A.J.); jie.zhao@delos.com (J.Z.); paigemp@umich.edu (P.P.); shaun.lyz@delos.com (S.L.); bauer.brent@mayo.edu (B.A.B.); 2 Delos Labs, Delos Living, New York, NY 10014, USA; 3 Mayo Clinic, Rochester, MN 55902, USA

**Keywords:** dynamic lighting, healthy building, intelligent control, office occupants, well-being, experience

## Abstract

As a critical factor in the built environment, lighting presents considerable influence on occupants. Previous research across static lighting conditions has found that both illuminance and correlated color temperature (CCT) affect occupants’ physiological and psychological functioning. However, little research has been conducted on the non-visual impacts of dynamic lighting with daily variation in illuminance and CCT levels. The purpose of this study is to better understand the impact of dynamic lighting on office occupants’ health, well-being and experience at a living lab. Fifteen participants were recruited to work in three office modules for four months. Four lighting conditions were designed and implemented in this study, including two static lighting conditions and two dynamic lighting conditions with a specific predefined control scheme. A prototype lighting system with enhanced control capabilities was configured and implemented to ensure the desired lighting environment protocol. Both objective methods and subjective surveys were used to assess the behavioral and physiological outcomes of interest, including mental stress, sleep, productivity, satisfaction, mood, visual comfort and perceived naturalness. The results showed that the daytime behavioral impacts were either positive or mixed. Specifically, a significant alertness increase was observed in the afternoon, indicating a potential solution to reduce the natural feelings of sleepiness during the workday. There was also a marginal benefit for mood. The nighttime impacts include a significant decrease in perceived sleep quality and sleep time after subjects were exposed to dynamic lighting. No significant differences were observed for mental stress, productivity, visual comfort, or perceived naturalness. The findings present additional insights into the non-visual impacts of dynamic lighting and give recommendations for further investigations.

## 1. Introduction

As a critical factor in the built environment, lighting presents considerable influence on occupants in multiple ways. In addition to the well-established effects for vision facilitation, lighting has effects beyond vision that can impact occupants’ physiological and psychological functioning [1,2,3,4,5]. Applied lighting research on the effects of light or lighting on humans is inherently interdisciplinary and would need expertise in several fields such as psychology, physiology, photobiology, architecture, and building physics [6].

It has been found that the effects beyond vision are primarily mediated by the intrinsically photosensitive retinal ganglion cells (ipRGCs) that contain the photopigment melanopsin [7,8], i.e., ipRGC-influenced responses to light (IIL responses). The ipRGCs differ from the classical rods and cones in their inherent characteristics of spectral sensitivity [7,9]. Previous research into IIL responses indicates that both illuminance and correlated color temperature (CCT) affect people’s well-being, health, and performance [10]. Through interactions between ipRGCs and specific brain regions, which include the amygdala and hypothalamus, and specifically the suprachiasmatic nucleus and ventrolateral preoptic nucleus, light can impact circadian rhythms, alertness, cognition, heart rate, and emotional activity [7,11,12,13,14]. While more photons (i.e., higher illuminance levels) are essential to induce the photoreception of melanopsin in the ipRGCs, higher CCT levels can also lead to greater IIL responses as melanopsin is most sensitive to blue light with shorter wavelengths at approximately 460–480 nm [15,16,17,18]. In addition, it has been demonstrated that exposure to blue light, either at night [5,19] or during the day [20,21], has impacts on occupants’ physiological and behavioral outcomes.

With respect to physiological and behavioral functioning, one of the most fundamental ways in which light impacts behavior is by influencing the human sleep/wake cycle [7,22]. More specifically, light can reset the sleep/wake cycle through interactions with ipRGCs, the suprachiasmatic nucleus, and the pineal gland. It is found that exposure to blue light in the morning entrains the human sleep/wake cycle to synchronize to the natural 24 h light/dark, thereby permitting physiological functions, such as sleep, to occur at the optimal time of day [23]. However, bright light exposure at night, which is typical in indoor environments with static light settings, can become highly disruptive to sleep [24,25,26,27]. 

In addition to the influence on sleep, there is evidence that lighting can affect occupants’ performance and self-reported productivity [28,29,30]. This is related to the impact that illuminance and CCT levels have on occupants’ alertness and ability to concentrate [3,30,31,32,33,34], as well as sleep, which impacts subsequent performance and productivity the next day [35,36,37]. Moreover, illuminance and CCT can also affect occupants’ satisfaction with and comfort in the environment [30,38,39]. Improved satisfaction with environmental conditions is associated with improved job satisfaction [40], which is in turn associated with improved job performance [41,42].

Previous studies have also shown that lighting can impact the biological and psychological processes related to mental stress [43]. Stress is often experienced within an office environment and it can have harmful effects on an individual’s physical and mental health [44]. Work stress is commonly associated with situations where the demands of the job exceed the individual’s skills [45,46], but recent research suggests that the factors of physical environment such as light can also impact occupants’ stress levels and overall well-being [47,48]. 

It is important to note, however, that previous experiments varied illuminance and CCT across static conditions, which do not reflect daily variations in natural light. Dynamic LED lighting, on the other hand, has emerged as an innovative lighting solution that can vary the CCT and illuminance simultaneously throughout the day [43]. With the help of various types of advanced lighting technologies and control schemes, dynamic lighting is able to mimic the daily variations in natural light [49,50,51]. This may present many potential benefits to occupants, including increasing satisfaction, increasing perceived naturalness of electric lighting, improving alertness, performance, and synchronizing human circadian rhythms [52]. Importantly, dynamic variations in light may play a calming role, which may indirectly alleviate stress [53,54]. Given these findings, dynamic light may be a potential solution to decrease stress during the day in office workers, while maintaining productivity, and improving satisfaction and sleep quality. However, there are limited studies that have looked into the impacts of dynamic light on the above-mentioned human outcomes. 

Therefore, this study was designed to better understand the impact of dynamic LED lighting with daily variation in illuminance and CCT levels. The goal was to investigate a specific dynamic LED lighting control strategy and evaluate its impacts on the health, well-being and experience of the occupants in an office environment. We hypothesized that dynamic lighting would reduce stress while maintaining productivity in the office. Moreover, we predicted that introducing dynamic lighting during the day, when office workers may not necessarily have direct exposure to natural light, would improve alertness during daytime and the subsequent sleep quality during nighttime. In the secondary analyses, we examined whether dynamic lighting affects mood, satisfaction, visual comfort, and perceived light naturalness. This study aimed to offer additional insights for a better understanding of the impacts of dynamic lighting on office occupants.

## 2. Study Design and Methods

### 2.1. Overall Study Design

This study employed a within-subjects design, where participants experienced each experimental condition. Specifically, behavioral measures for each participant were compared across 4 different predefined lighting conditions. As the main hypothesis was that dynamic lighting would improve behavioral outcomes compared to static lighting, all subsequent analyses compared static lighting to dynamic within a specific country.

This study was conducted across a total of approximately 4 months. Each lighting condition was conducted continuously for 4 weeks. More specifically:

- 2 static lighting conditions that represent typical lighting settings of Japanese (JP) and U.S. offices (i.e., JP-T and US-T conditions);
- 2 dynamic lighting conditions with varying lighting CCT and illuminance (i.e., JP-D and US-D conditions);
- 2 weeks were assigned as the acclimation period at the beginning of this study;
- 1 additional week was assigned to handle the sleep/wake schedule changes due to daylight savings time.

**Figure 1:**
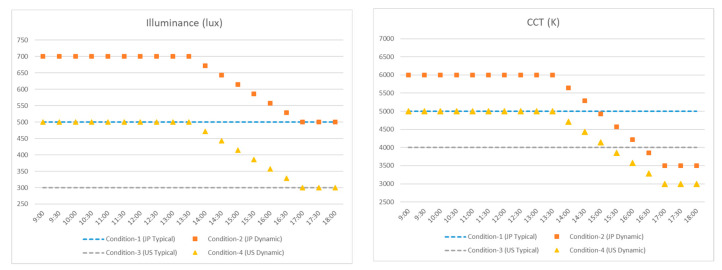
Lighting condition design in this study.

Figure 1 describes the different experimental lighting conditions during office hours. The settings were defined based on the light fixture capability, industry interest, and several guidelines including:

- Condition 1 (JP-T) represented typical lighting conditions in Japanese offices, i.e., 500 lux/5000 K at the horizontal desk level.
- Condition 2 (JP-D) was the dynamic lighting profile for Japanese offices. It varied in reasonable Japanese lighting ranges, i.e., the illuminance falls between 500 and 700 lux and CCT falls between 3500 and 6000 K.
- Condition 3 (US-T) represented typical lighting conditions in US offices, i.e., 300 lux/4000 K.
- Condition 4 (US-D) was the dynamic lighting profile for U.S. offices. It varied in reasonable U.S. lighting ranges, i.e., the illuminance falls between 300 and 500 lux and CCT falls between 3000 and 5000 K.
- Dynamic lighting profiles (i.e., JP-D and US-D) presented increased illuminance and CCT levels in the morning compared to the corresponding static lighting level.
- Dynamic lighting profiles presented gradually decreased illuminance and CCT levels in the afternoon to mimic the natural daylighting variations
- JP-D and US-D shared the same magnitude in illuminance variation, i.e., to present a constant shift between them over time
- JP-D and US-D shared the same CCT variation trends that can create similar recognized color change over time.

### 2.2. Office Configuration

**Figure 2:**
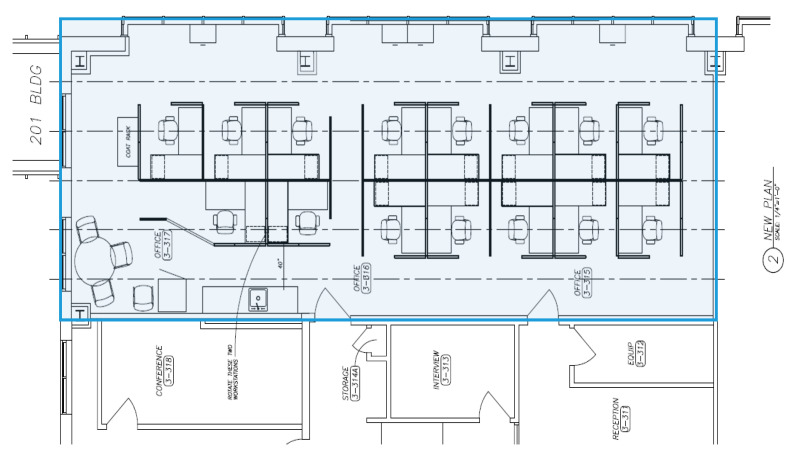
Floor plan of the study modules and desk layout.

This study was conducted at a living lab that is explicitly designed to investigate the interactions between humans and the indoor environment [55,56]. Figure 2 shows the floor plan of the study modules and desk layout. Three modules in the lab were combined into a 124 m^2^ open office (19.1 m length, 6.5 m width, and 2.6 m height) to conduct this study. 

The modules implemented various building technologies such as sheer shades and electrochromic glass control to isolate the outdoor lighting conditions and support the electrical lighting system to achieve the desired indoor lighting environment. More specifically, the shading and glass settings were carefully configured to reduce the impact of natural lighting on the indoor lighting environment while still providing occupants view access outdoors to avoid possible reductions to their productivity [57].

### 2.3. Lighting System Design

**Figure 3:**
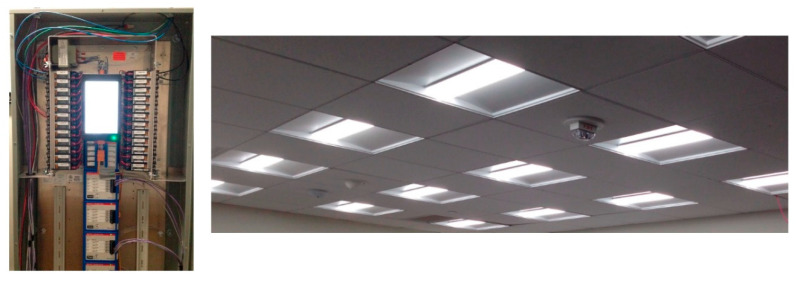
Photo of the lighting system controller (**left**) and lighting luminaires (**right**).

A prototype lighting system developed by Universal Lighting Technologies and Douglas Lighting Controls (owned by Panasonic) was selected and installed in the modules to create reliable dynamic lighting control for this study. As shown in Figure 3, the system included an advanced lighting controller unit that can provide programming capabilities and an array of LED luminaires that can vary both the illuminance and CCT. 

In order to ensure sufficient control flexibility to satisfy study requirements, the luminaires layout was carefully designed and separated into 15 control groups, each of which can be configured with various settings. The layout of the desks was also carefully designed to benefit the lighting spatial uniformity across all the desks. Once the luminaire and desk layouts were confirmed, initial lighting tests were conducted at the desk level to identify the control settings at different time of the day. The settings were then programmed in the advanced lighting controller to create the desired lighting profiles for all the lighting conditions.

### 2.4. Participants

#### 2.4.1. Participant Exclusion Criteria

Fifteen participants (Male = 9, M_age_ = 38) were recruited across various work units at a large midwestern medical institution. Individuals with a history of the following were excluded in the participant selection:

- diagnosed sleep disorders,
- severe vision problems,
- sensitivity to light resulting in headaches or seizures,
- cognitive abilities interfering with typical office work,
- physical disabilities interfering with typical office work,
- severe mood disorders, and
- drug or alcohol dependency.

Individuals who were in their office for less than 50% of the time or participating in a performance improvement plan were not considered for this study. Finally, due to the lengthy nature of this study, women who were pregnant or intending to become pregnant during this study were not considered for this study. Eligible participants underwent a screening process that included a medical chart review, including OTC medication assessment and travel that was more than two time zones away throughout this study. Based on the chart review, the participants who did not meet the requirements were excluded. All participants gave written informed consent before the start of this study. This study followed a standard subject recruitment procedure and did not have compulsion in the selection of participants. The study design was reviewed and approved by the institutional review board (IRB) at a well-recognized non-profit academic medical center.

One of the participants traveled internationally (>2 time zones) for 7 days during the middle of a condition (US Dynamic). Sleep data for those travel dates and the following week were excluded from the final analysis to control for abrupt changes to their sleep schedule due to jet lag. At the start of this study, all participants completed validated questionnaires establishing demographics, baseline perceived sleep quality, and chronotype. The final sample size was predominantly white and middle class. With respect to chronotype, participants categorized themselves as mostly moderate morning and intermediate types. Finally, with respect to baseline perceived sleep quality, participants rated themselves as slightly above the cut-off score of 5, indicating slightly worse sleep quality (<5 indicates good sleep quality). 

#### 2.4.2. Demographic Information

For a breakdown on the group’s demographics, please refer to Table 1.

#### 2.4.3. Chronotype

Given that a person’s chronotype can impact the exposure to light a person receives [58,59] and may even influence their behavioral response to light [60], we measured participants’ chronotype using the Morning–Eveningness Questionnaire (MEQ) [25,61] prior to the start of the experiment, as shown in Table 2.

The MEQ is a well-validated and widely used self-report questionnaire developed to measure whether an individual’s circadian rhythm produces peak alertness in the morning, evening, or in between. It has a 19-item scale, where participants answer multiple choice questions about their sleep/wake habits. Responses are coded to determine whether that individual is more active in the morning or evening. In the current study, the MEQ was administered prior to the start of the experiment to measure the chronotype of participants. Scoring of MEQ used parameters set by a more recent validation study [62] accounting for middle-aged individuals, which more accurately reflects the likely demographics of the current study.

### 2.5. Environmental Measurement Methods 

As the primary environmental factor in this study, lighting condition was carefully configured and measured in both the study design and operation phases. In the study design phase, the lighting system was configured based on the spatial distribution measurement. In the study operation phase, the actual lighting environment was measured continuously to evaluate the temporal lighting variation caused by various types of interferences. The non-visual impact of the dynamic lighting was also theoretically analyzed using the measured lighting SPD data. Additionally, other indoor environment parameters as well as the building system operations.

**Figure 4:**
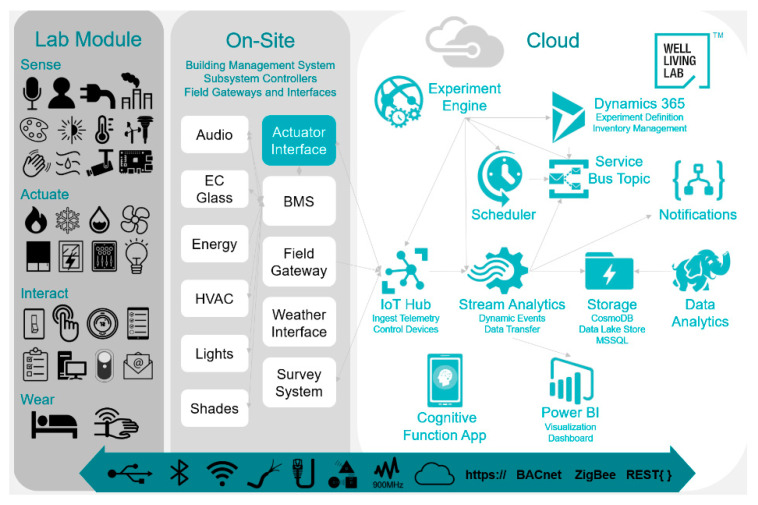
Schematic chart of the IoT system for environmental control and data collection.

The measurement was conducted using the lab’s environmental monitoring and control platform, equipped with an advanced building automation system (BAS), sensor technology, and internet of things (IoT) capabilities. The schematic chart of the IoT system can be found in Figure 4 and additional information can be found in our previous publications [55,56]. 

**Figure 5:**
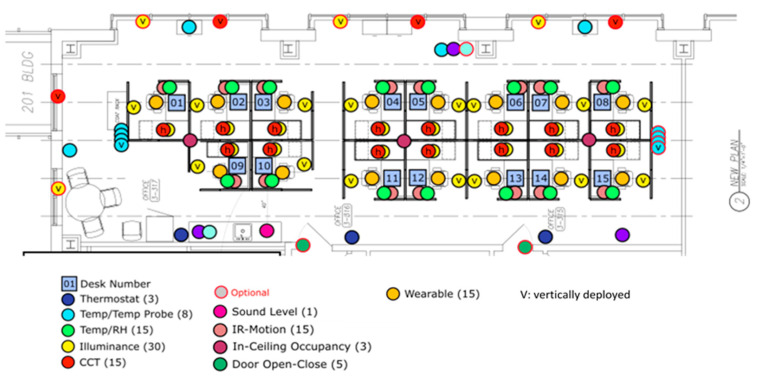
Sensor deployment for environmental measurement.

More specifically, 144 environmental sensors of the following types were deployed in the modules following the sensor deployment map shown in Figure 5. More details on the sensor selection and configuration can be found in [63].

- Lighting environment: wireless real-time sensors (Lux1000 and Color Lux1000, Wovyn LLC, Heber City, UT, USA) were deployed at the horizontal desk surfaces and vertical window surfaces to measure the illuminance and CCT.
- Thermal environment: wireless real-time sensors (Monnit Corp., South Salt Lake, UT, USA) were deployed at each desk to measure the thermal environmental parameters that may affect occupant performance and satisfaction, including temperature, relative humidity, and radiant temperature.
- Acoustic environment: a real-time acoustic analyzer (XL2 audio and acoustic analyzer with M2211 microphone, NTi Audio Inc., Schaan, Liechtenstein) was deployed to measure the equivalent sound level for acoustic evaluations.
- Indoor air quality: measure the module-level ventilation rates for indoor air quality evaluations.
- Building operations: measure operational parameters of the building system, including the shades operation and electrochromic glass control.

### 2.6. Behavioral Outcome Measurement Methods

This study aimed to measure the efficacy of using dynamic light to reduce stress in the office environment while also maintaining productivity and improving sleep quality. Changes in these primary behavioral outcomes of interest were measured using objective and/or subjective approaches. Physiological stress responses were measured using the Empatica E4, a non-intrusive multisensory monitoring device, whereas sleep behaviors were measured using the Early Sense contact-free sleep system. Assessments with subjective scales were administered daily and monthly through Qualtrics (Qualtrics, Provo, UT, USA) on their phone or computer. The design of the subjective surveys is described below in each subsection.

The sleep diary and sleep sensor data were collected daily from all participants. Participants completed short surveys five times a day to measure perceived stress, alertness, and mood. In addition, participants completed additional surveys at the end of each workday to measure perceived productivity, satisfaction with the environment, and visual comfort. Finally, participants received surveys at the end of each condition (monthly) to measure changes in perceived sleep quality, perceived stress (work-related and overall), and perceived naturalness of the light. 

#### 2.6.1. Stress

Previous studies have shown that both the illuminance and CCT influence heart rate and core body temperature but not cortisol (stress hormone) concentration [4,43,64]. Heart rate variability [65,66], skin temperature [67], and electrodermal activity (EDA), also known as galvanic skin response (GSR) [65], have been correlated with mental stress. In order to assess physiological and psychological indices of stress, we used the following objective and subjective measures.

- Objective physiological measurements

**Figure 6:**
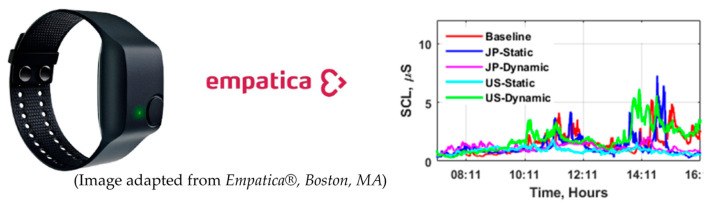
Photo of Empatica E4 and sample data for continuous stress measurements.

Built-in sensors in smartphones and wearable devices have become an increasingly popular way to detect stress features in a non-intrusive manner. For this study, we continuously monitored the psychophysiological metrics related to sympathetic arousal states Empatica E4, as shown in Figure 6. The Empatica E4 is a non-intrusive wireless multisensory device for real-time data acquisition. It is equipped with four embedded sensors, including EDA, photoplethysmograph, 3-axis accelerometer, and skin temperature allowing the monitoring of different psychophysiological body responses [68].

Analysis of the EDA signals retrieved from the wearable device was performed. EDA is a measure of the changes in conductance of the skin due to thermoregulation or physiological arousal and it is directly related to the amount of sweat secretion by the sweat glands [69,70]. The EDA signal is characterized by two main components. The first component is skin conductance level (SCL), a slowing changing part that reflects the tonic level of electrical conductivity of the skin. The second component is skin conductance response (SCR), a phasic component that reflects fast changes in the EDA signal. SCRs can further be classified in specific SCRs, the signals of which are characterized by a fast change by a stimulus [71]. Non-specific SCRs (NS-SCRs) occur in the absence of an external stimulus. Both SCL and SCR are sensitive to stress-related events [72,73]. For analysis of the EDA data, a continuous decomposition analysis (CDA) was conducted to extract the tonic component of the skin conductivity signal, SCL and calculate NS-SCRs characteristics given the relatively long-term EDA measures. In this study, differences in tonic and phasic retrieved information for each experimental condition including baseline were evaluated using repeated-measures ANOVAs and independent group *t*-tests.

- Daily subjective stress measurement

Participants were asked to rate the intensity of their stress using a 7-point Likert scale, 1 (not at all) to 7 (extremely), at five different time points (9:00 AM, 11:00 AM, 1:00 PM, 3:00 PM, and 5:00PM). The specific time points were selected to reflect intervals before, during, and after illuminance and CCT changes in the dynamic lighting conditions. Moreover, participants were asked to rate their level of physical activity during the five different time points as light (walking slowly, sitting using a computer or standing light work), moderate (walking briskly) and vigorous (jogging, carrying heavy loads), as this could impact stress levels.

- Monthly subjective stress measurement

The perceived stress scale (PSS-10) was used to assess the degree to which situations in participants’ lives are appraised as stressful [74]. PSS-10 is one of the most widely used psychological instruments to measure levels of experienced stress. It consists of 10 individual items related to participants’ feelings of how unpredictable, overloaded or uncontrollable they find their lives. It requires the person to fill out how often they have felt that way on a scale from 0 (never) to 5 (very often). The PSS-10 was administered monthly at the end of each lighting condition.

In addition, we asked participants to fill out a monthly questionnaire about the levels of job stress experienced during each lighting condition. The job stress scale is a 5-item index focused on worker’s experience of stress in the work environment and questions are descriptive of the emotional states experienced by employees. The scale has been shown to have good reliability [75].

#### 2.6.2. Sleep and Circadian Rhythms

Previous lighting interventions have been found to impact participants’ circadian systems and thus their subsequent sleep quality after office hours. In this study, participants’ sleep was assessed based on the measurement using the following objective and subjective approaches.

- Objective real-time sleep tracking

**Figure 7:**
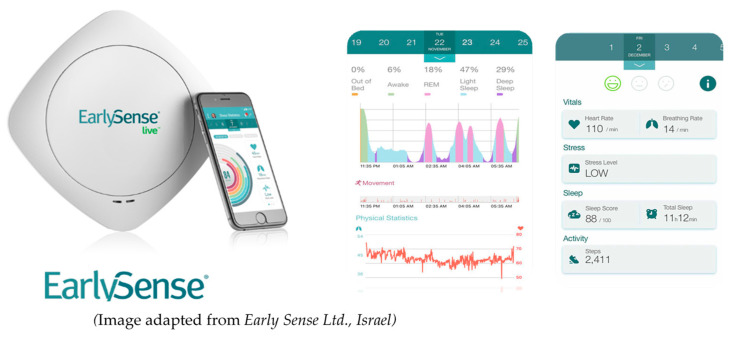
Early Sense for continuous real-time sleep tracking.

Changes in sleep across conditions were measured using a contact-free system based on a piezo-electric sensor called Early Sense Live, as shown in Figure 7. Heart rate, respiration rate, and movement were collected and analyzed to calculate various sleep parameters. The Early Sense Live has been validated by the manufacturer against polysomnography, the sleep measurement gold standard [76].

Participants received the device along with instructions to install the sensor under their mattress at home. An automatic sleep algorithm developed by Early Sense was used to classify wake, sleep, and sleep stage in 30 s increments. These were combined across the night to calculate the following sleep parameters: total sleep time (TST), sleep onset (when someone fell asleep), sleep onset latency (how long it took someone to fall asleep), bedtime (when someone got into bed), wake time (time someone woke up in the morning), wake after sleep onset (WASO-number of times awakened), time getting out of bed, time in each sleep stage (light sleep, deep sleep, rapid eye movement-REM sleep), and sleep efficiency (total sleep time/total time in bed). Finally, to assess any shifts in individuals’ biological rhythms due to their daytime light exposure, the midpoint of sleep was calculated. Note that only weekday data were analyzed due to insufficient data during the weekends. Nights with irregular sleep patterns of total sleep times <2 h or >12 h were excluded from the analysis.

- Daily subjective measurement—sleep diary

To corroborate and validate sleep measurements obtained from the Early Sense Live, participants were asked to keep a sleep diary where they reported bed and wake times, total sleep time, perceived nighttime awakenings, daytime naps, bedtime habits, and daily caffeine intake. In addition, to measure participants’ perceptions of how well they slept, they were asked rate their sleep quality on a 5-point Likert scale—1 (very bad) to 5 (very good). Diaries were administered through survey software for all participants for the duration of this study on both work days and non-work days. As with the objective sleep data, only weekday data from sleep diaries were analyzed.

- Daily subjective measurement—alertness

At the same time points as the daily subjective stress measurements (see Section 2.6.1), participants rated their alertness using the Stanford Sleepiness Scale (SSS), a well-validated psychometric tool for populations aged 18 and older [77]. The SSS is a one-item self-report questionnaire which measures sleepiness throughout the day. Participants rated alertness on a 7-point Likert scale—1 (feeling active, wide awake, alert) to 7 (no longer fighting sleep, sleep onset soon).

- Monthly sleep quality measurement

To measure the lasting impact of the different lighting conditions on perceived sleep quality, participants completed the Pittsburgh Sleep Quality Index (PSQI) [78]. The PSQI is a self-report questionnaire developed to assess sleep quality over a 1 month interval. It consists of 19 individual items which are organized into 7 components that subsequently produce one global score of sleep quality. The PSQI is well-validated in both clinical and research settings and is intended to be used with ease across different populations. In this study, the PSQI was administered monthly at the end of each lighting condition.

#### 2.6.3. Productivity 

Participants were given a set of questions from the Cost-effective Open-Plan Environments (COPE) survey, a widely used questionnaire adapted by the Center for Building Performance and Diagnostics at Carnegie Mellon University to capture satisfaction at a given moment [40,79]. The survey was adapted in this study, including only perceived daily productivity, satisfaction with the environment, and the extent to which participants believe the environment is impacting their productivity. The COPE was administered at the end of each workday through Qualtrics survey software.

#### 2.6.4. Secondary and Exploratory Outcomes 

This study also aimed at measuring the efficacy of using dynamic light to improve satisfaction, comfort, perceived naturalness of electric lighting, and mood (positive affect and negative affect). Changes in these outcomes were measured and compared across conditions in exploratory analyses.

- Satisfaction

Participants’ satisfaction with lighting conditions, other environmental conditions, and with the overall environment was measured using the COPE survey on a 7-point scale.

- Visual comfort

Participants’ visual comfort was measured using the Headache and Eye Strain Scale (H and ES) [30], assessing the impact of the different dynamic lighting conditions on daily visual comfort compared to the corresponding static condition. The scale consists of items which assess the severity of symptoms such as eye fatigue, blurred vision, and headache. This scale was administered at the end of each workday through survey software. Possible scores for individual items range from 1 (none) to 4 (severe).

- Mood

Participants’ mood was measured using the Positive Affect Negative Affect Schedule (PANAS) survey [80]. Mood and, more broadly, a person’s affective state—which can encompass their experience of emotional states, feelings, and mood—is typically defined on two dimensions which function independent of each other. Mood may also affect occupants’ stress levels. Positive affect (PA) describes a person’s enthusiasm and energy level and can also be used to quantify a person’s ability to cope with adversity. Negative affect (NA), on the other hand, may indicate a person’s distress or anxiety. PA and NA were measured through the PANAS survey, which consists of 10 positive and 10 negative adjectives for feelings. For each of the 20 emotions, participants are asked to rate the extent to which they experience that emotion on a 5-point Likert scale. Participants were asked how they felt at the same time points as when they were asked to report their alertness and subjective stress response.

- Naturalness

With variant illuminance and CCT levels, dynamic lighting may appear more natural. Participants’ perceptions of the naturalness of light were measured using the monthly survey approach. A light naturalness scale designed in [52] was used to assess participants’ perception of how natural the light is. Participants rated the naturalness at the end of each workday on a 7-point Likert scale (very unnatural to very natural). This scale was administered monthly, at the end of each lighting condition, through survey software.

### 2.7. Statistical Analysis Method

Linear mixed-effects analyses were performed using the lme4 (Version 1.1-15) [81] package in R (Version 3.4.1, R Foundation for Statistical Computing, Vienna, Austria) [82] to measure the relationship between each dynamic lighting condition and its corresponding static condition and the previously defined outcomes, specifically, stress, perceived productivity, sleep, mood, alertness, environmental satisfaction, and perceived naturalness of lighting.

Mixed-effect analyses are a standard approach in the behavior science area and have been proven strong and effective in analyzing similar occupant outcomes. These allowed researchers to model the variation in how each participant reacted to each lighting condition. For each of the outcomes mentioned above, environmental condition (static lighting, dynamic lighting) was a fixed effect. For perceived daily stress, monthly ratings of job-related stress and perceived stress were used as covariates to measure their impact on daily stress measurements. Intercepts for the random effect of participants and by-participant random slopes for the effect of condition were included in each model. Satterthwaite’s approximation using the lmerTest package (Version 2.0-36) [83] was used to obtain significance values for parameter estimates. Confidence intervals for fixed-effect estimates were obtained using the effects package (Version 4.0-1) [84]. Statistical significance was defined as two-sided *p*-values < 0.05. 

## 3. Lighting Configuration and Environmental Data Analysis Results

### 3.1. Evaluation of the Lighting Settings for This Study 

The illuminance and correlated color temperature (CCT) at all desks were measured to support the lighting controller configuration, as well as the evaluation of the desk layout and luminaire layout design for a uniform light environment.

The spatial distributions analysis results showed that the standard deviation of the illuminance/CCT measurements was less than 20 lux/40 K for all conditions, indicating a uniform illuminance/CCT distribution. The difference between the average measurement and the design value was less than 10 lux/100 K, indicating a satisfactory lighting controller configuration.

### 3.2. Analysis of the Actual Lighting Environment during This Study

To ensure a consistent lighting environment during this study, the actual lighting environment was monitored continuously using wireless lighting sensors in the study operation phase. Compared to the measurements performed during the design phase, the actual lighting environment measurement needed to account for various types of interferences including daylighting, occupant movement, and unexpected lighting sources such as screens. The measured data can help to understand the magnitude of the interference, as well as to detect any significant deviations from the design lighting profiles. 

#### 3.2.1. Temporal Illuminance Variations 

**Figure 8:**
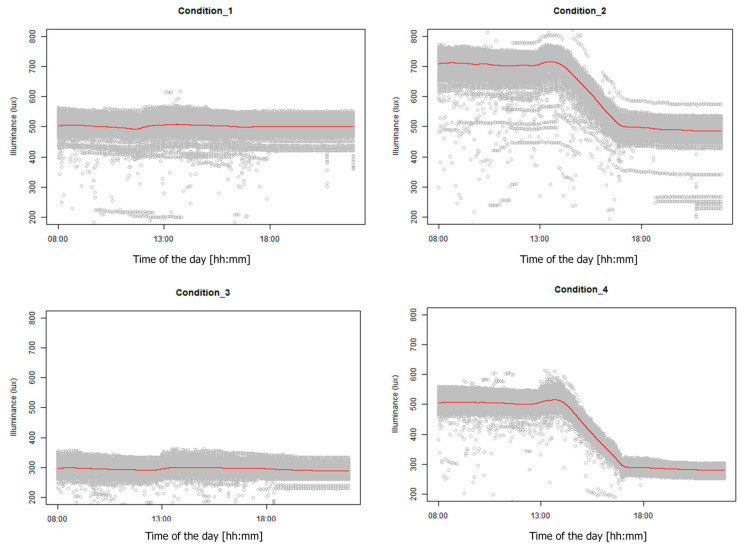
Daily temporal illuminance variations in all lighting conditions (Condition_1: JP-T; Condition_2: JP-D; Condition_3: US-T; Condition_4: US-D).

During this study, 15 illuminance sensors were placed at the desk surfaces to measure the temporal illuminance variations. The data were collected once per minute and we calculated the 15 min average for the analysis and plotting, as shown in Figure 8. The figure includes the data for all desks throughout the study period. Grey dots represent measurement data, while the red line represents the average value at that minute. It can be observed that the variations caused by the interference (i.e., the width of the grey area) is less than 50 lux for the most of the time, and the red line closely follows the designed lighting profiles. There is an increase at approximately 1:00–2:00 PM across all conditions due to external solar radiation, but the slight increase does not significantly impact the average illuminance to the extent that it no longer falls within the acceptable range. The transition period (i.e., 2:00–5:00 PM) profiles in the dynamic conditions are reasonably smooth, indicating satisfactory configuration and dynamic illuminance control capabilities of the installed lighting system.

#### 3.2.2. Temporal Lighting Correlated Color Temperature Variations 

**Figure 9:**
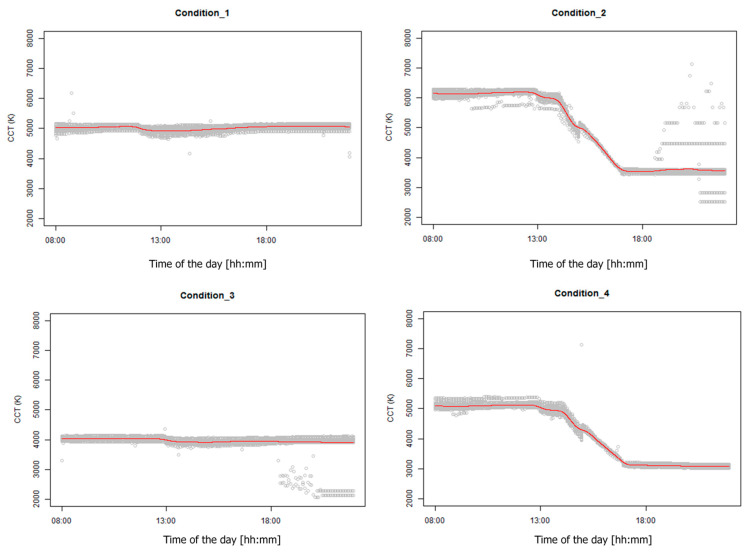
Daily temporal CCT variations in all lighting conditions (Condition_1: JP-T; Condition_2: JP-D; Condition_3: US-T; Condition_4: US-D).

A total of 15 CCT sensors were placed at the side desks to measure the temporal CCT variations, as summarized in Figure 9. Compared to the illuminance data discussed in the above section, the grey area size for CCT is smaller, indicating relatively less interference on the CCT in this study. The red line is close to the designed lighting profiles, and the transition period (i.e., 2:00–5:00 PM) profiles in the dynamic conditions are reasonably smooth. This indicates satisfactory configuration and dynamic CCT control capabilities of the installed lighting system. There is a decrease between 1:00 and 3:00 PM because of the external solar radiation and tinted glass, but the decrease, again, falls within an acceptable range of deviation. 

The temporal illuminance and CCT variation data proved the efficacy of the designed approach, i.e., integrated operation of the sheer shades and electrochromic glass, to ensure the desired lighting environment.

### 3.3. Theoretical Quantification of the Non-Visual Impact of the Dynamic Lighting

#### 3.3.1. Lighting Property Data Analysis

**Figure 10:**
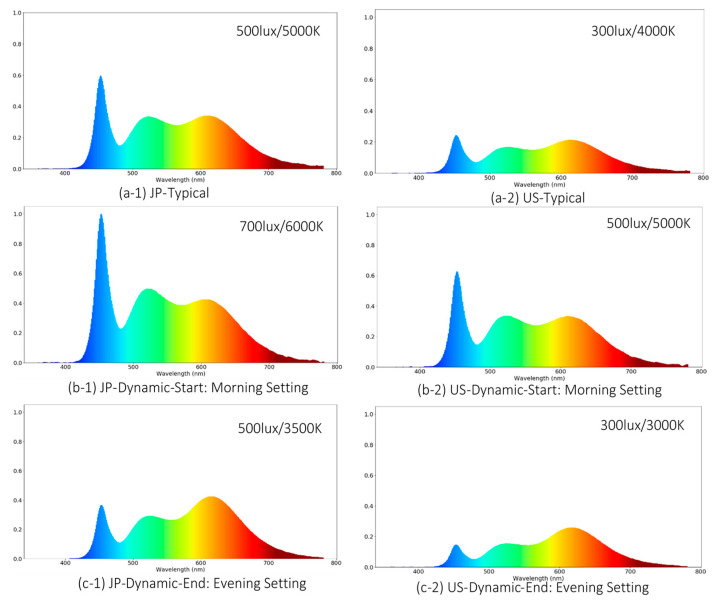
Normalized spectral power at representative lighting configurations.

In addition to the illuminance and CCT measurement, the spectral power distribution (SPD) data were measured in the lighting configuration phase. This was performed at each desk surface for all the representative lighting conditions. As shown in Figure 10, the blue band of the installed LED lighting luminaires peaks at approximately 450 nm. In the current lighting literature, there are multiple theories on the physiological effects of lighting. While some researchers consider 430–500 nm as the bioactive blue range [85], others believe that the range around 460 nm at which circadian stimulus peaks [86] and the 480 nm at which melanopsin SPD sensitivity peaks [87] should be paid more attention. In a recent study by Martin Moore-Ede et al., a noval human Circadian Potency spectral sensitivity curve is derived with a peak at 477 nm [88]. From the SPD measurements, it can be seen that the luminaires implemented in this study can considerably change the magnitude of spectral power within the bioactive band (i.e., 430–500 nm) across different lighting conditions, but the difference at the 460 nm (circadian stimulus peak) band or 480 nm (melanopsin sensitivity peak) band is relatively less considerable. 

#### 3.3.2. Non-Visual Impact Estimation and Analysis

Based on the measured SPD data shown in Section 3.3.1, the non-visual impacts of the dynamic lighting on the human circadian rhythm were theoretically quantified using two state-of-the-art approaches. One is the Equivalent Melanopic Lux (EML) approach based upon a melanopsin-only spectral weighting of irradiance, and the other one is the circadian stimulus (CS) approach based on the circadian stimulus [89,90,91].

**Figure 11:**
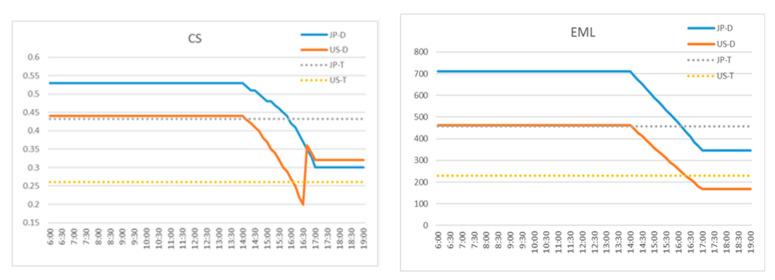
Non-visual impact estimation using CL and EML approaches.

As can be seen in Figure 11, the CS and EML profiles in the dynamic lighting conditions have similar trends to the illuminance and CCT profiles in the corresponding lighting condition. In addition, a remarkable difference can be observed between the dynamic and static lighting conditions. Although these results cannot directly indicate the impacts of dynamic lighting on the investigated human outcomes in this study, they provide valuable insights to understand the magnitude of the impact on human circadian rhythm.

### 3.4. Evaluation of Indoor Non-Lighting Environment and System Operations

In addition to the lighting environment measurement, the non-lighting environment was also comprehensively measured, including the thermal environment, acoustic environment, and the indoor air environment. The system operations were also measured to detect any operational faults of this study. It was found that during the study operation these factors were in the normal range for a typical office building [92]. For more details on the measurement and assessment of these parameters in a living lab, please refer to [63].

## 4. Behavioral Data Analysis Results

For each of the reported results, dynamic lighting was compared to static lighting within a specific country (i.e., US-T vs. US-D conditions; JP-T vs. JP-D conditions). 

### 4.1. Mental Stress Analysis

#### 4.1.1. Subjective Stress Analysis

The subjective stress analysis found no significant differences between static and dynamic lighting in either of the country-specific conditions. This means that when participants were asked to comment on their overall stress at different time points during the day, or the overall stress and job-related stress across the entire lighting condition, they perceived no significant difference after being exposed to dynamic lighting compared to when they were exposed to the corresponding static lighting. 

#### 4.1.2. Objective Stress Analysis

Analysis of the EDA signals retrieved from the wearable device was performed using MATLAB^®^ (MathWorks, Natick, MA, USA) and Ledalab^®^. The skin conductivity level (SCL) measured during a fixed time period for 12 study participants at all study conditions were extracted and compared. There were three participants excluded from the SCL fixed time analysis given the lack of consistency in the times spent in the office across conditions. Comparisons of SCL at fixed times between the country-specific conditions revealed no significant differences. 

In order to compare the amount of phasic activity across the different conditions, the mean NS-SCR amplitudes were computed from the CDA analysis for all subjects without restricting the data to specific time periods. Comparisons of NS-SCR amplitude values within subjects for static vs. dynamic conditions within a specific country were conducted. These results indicate that stress or arousal events were on average the same for static vs. dynamic conditions for both countries. 

The average phasic driver which results from sudomotor nerve activity across the different experimental conditions was also quantified as a measure of the intensity of the phasic responses. Similar to the NS-SCR amplitude analysis, the average phasic driver was measured for all subjects without restricting the data to specific time periods. These results indicate that the sympathetic activity related to stress or other arousal events was on average the same for static vs. dynamic conditions for both countries.

Overall, there was no difference in the sympathetic activity related to stress or arousal across static and dynamic conditions for both countries, based on the objective stress analysis using EDA-extracted features. This finding aligns with those obtained in the subjective stress analysis.

### 4.2. Sleep Analysis

#### 4.2.1. Perceived Sleep Quality

- Daily sleep quality

Participants’ daily perceived sleep quality was compared between static and dynamic lighting in either of the country-specific conditions, based on the measurements of participants’ daily diary responses. There was no significant difference in sleep quality between static and dynamic lighting in the US-specific lighting conditions. 

There was, however, a significant decrease in perceived sleep quality in the JP-D lighting condition compared to the JP-T condition. Additionally, when accounting for week number in our analyses, we found that the perceived sleep quality became progressively worse as the JP-D dynamic condition progressed. One explanation for this decrease could be that as the condition progressed, time in deep sleep—a restorative sleep stage—decreased, indicating that although people sleep the same amount, it was not as restorative and restful.

- Monthly sleep quality

There were no significant differences in sleep quality, as measured by the PSQI, between static and dynamic lighting in either of the country-specific parameters. This means that when participants were asked to evaluate their overall sleep quality across the month, they perceived their sleep quality to be approximately the same after being exposed to dynamic lighting during the day as it did when they were exposed to static lighting. This was observed in the two country-specific conditions. 

#### 4.2.2. Nighttime Sleep Analysis

Changes in the following nighttime sleep parameters across conditions were analyzed based on the continuous objective sleep data measured by Early Sense Live as well as the subjective data from the sleep diary. Table 3 lists the results for continuous objective sleep measurements.

- Total sleep time

There was a significant difference in total sleep time between US-T and US-D lighting conditions. Specifically, participants slept approximately 32 min less after being exposed to dynamic lighting compared to static lighting (*p* < 0.001). Within the JP-specific lighting conditions, there were no significant differences in total sleep time between static and dynamic lighting.

- Sleep onset

There was a significant difference in sleep onset in the US-specific lighting conditions. Specifically, participants fell asleep approximately 20 min later after being exposed to dynamic lighting compared to static lighting (*p* = 0.035). Within the JP-specific lighting conditions, there were no significant differences in sleep onset time between static and dynamic lighting, which means that participants went to bed at approximately the same time.

- Time in deep sleep

There was no significant difference in the amount of time participants spent in deep sleep in the US-specific lighting conditions, which means that participants spent approximately the same amount of time in deep sleep in both static and dynamic conditions. In the JP-specific conditions, however, the time spent in deep sleep progressively decreased throughout the dynamic condition such that participants received approximately 24 min less deep sleep (*p* = 0.054).

- Time in light sleep

There was a significant difference in the amount of time spent in light sleep in the US-specific lighting conditions. Specifically, participants slept approximately 20 min less after being exposed to dynamic lighting compared to static lighting (*p* = 0.022). Within the JP-specific lighting conditions there were no significant differences in light sleep between static and dynamic lighting.

- Other sleep parameters

Analysis was also conducted for several other sleep parameters, including sleep onsite latency, sleep efficiency, wake after sleep onset, and time in REM sleep. There were no significant differences in these parameters between static and dynamic lighting in either of the country-specific parameters. 

#### 4.2.3. Alertness—Perceived Feeling of Sleepiness during Daytime

Within the US conditions, there were no significant differences in alertness between the static and dynamic lighting conditions throughout the day. Within the JP conditions, however, alertness increased at 1:00 PM in the afternoon in the dynamic lighting condition, compared to that under static lighting condition (*p* = 0.015). Results for daily alertness measurement are summarized in Table 4.

### 4.3. Productivity Analysis

There were no significant differences in perceived productivity, as measured by the COPE, between static and dynamic lighting in either of the country-specific parameters. This means that when participants were asked to evaluate their productivity across the month—as well as their perceptions on how the environment may have contributed to their productivity—they perceived no difference after being exposed to dynamic lighting during the day compared to when they were exposed to static lighting. This effect was observed in the two country-specific conditions. 

### 4.4. Secondary Measures Analysis

#### 4.4.1. Satisfaction

- Satisfaction with lighting

There were no significant differences in satisfaction with lighting between static and dynamic lighting in either of the country-specific parameters. This means people felt equally satisfied with the lighting conditions in both the static and dynamic conditions. This effect was observed in the two country-specific conditions.

- Satisfaction with other environmental parameters

Within the US condition, there were no significant differences between the static and dynamic lighting conditions in satisfaction for other environmental parameters, including the air quality, acoustic conditions, visual privacy, etc. This means that participants were equally satisfied with their overall environment in both lighting conditions. 

Within the JP condition, interestingly, even though the only environmental parameter that changed across this study was the indoor lighting levels, participants felt more satisfied with the background noise (*p* = 0.024) and acoustic privacy (*p* = 0.027) in the dynamic condition compared to the static condition. In addition, participants felt marginally more satisfied with cleanliness of the work area (*p* = 0.054) and air movement in the office (*p* = 0.078) in the dynamic condition compared to the static condition. Previous research [93] demonstrates that people may view their environment holistically and perceive changes or improvements in certain aspects of their environment (i.e., better air quality) when modifications in other factors have occurred (i.e., room becomes brighter due to increased access to windows). However, as seen above, participants did not perceive any improvement in their lighting environment, which makes this explanation unlikely, as previous work shows that participants still notice what was actually altered. In addition, during the JP-T condition, minor construction occurred in some of the nearby modules, which concluded towards the beginning of the dynamic condition. Therefore, these ratings may reflect participants’ satisfaction with the conclusion of the nearby construction. 

#### 4.4.2. Visual Comfort

There were no significant differences in visual comfort between static and dynamic lighting in either of the country-specific parameters. This means people felt no negative effects (symptoms of headache or eyestrain) for the different lighting conditions. This effect was observed in the two country-specific conditions. In both country conditions, participants indicated that they experienced more glare from the overhead lighting in the dynamic condition, but this discomfort was relatively minor.

#### 4.4.3. Mood Analysis

- Positive affect

In the US-specific lighting conditions, there was a marginal decrease in positive affect (PA) at 5:00 PM in the afternoon in the dynamic condition compared to the static condition (*p* = 0.073). In the JP conditions, PA marginally increased at 3:00 PM in the dynamic condition compared to the static condition (*p* = 0.079). These marginal differences indicate that although a difference was present, it did not quite meet the requirements for statistical significance. Detailed results for the PANAS mood measurements (PA) are listed in Table 5.

- Negative affect

There were no significant differences in negative affect (NA) between static and dynamic lighting in either of the country-specific parameters. This means the frequency at which people felt negative emotions during the day did not change in both the static and dynamic conditions. This effect was observed in the two country-specific conditions. 

#### 4.4.4. Perceived Naturalness of the Light

There were no significant differences in the perceived naturalness of the light between static and dynamic lighting in either of the country-specific parameters. This means that when participants were asked how natural they found the dynamic lighting in the office compared to the static office lighting, they perceived the naturalness to be approximately the same. This effect was observed in the two country-specific conditions. 

## 5. Discussions

### 5.1. Daytime Impacts

Test results showed that the behavioral impacts presented by dynamic lighting during daytime were either positive or mixed. Specifically, a significant alertness increase was observed at 1:00 PM in the afternoon in the JP-D condition compared to the JP-T condition. This time point represents a cognitive dip, where, due to circadian patterns, most people feel sleepier and less alert. Therefore, this finding indicates that dynamic lighting may be an effective solution to reduce the natural feelings of sleepiness during the workday. 

It is also observed that a marginal increase in positive affect (PA) in mood occurred at 3:00 PM in the JP-D condition, while a marginal decrease in PA occurred at 5:00 PM in the US-D condition. This somewhat dichotomous difference could be potentially explained by differences in illuminance and CCT between country-specific conditions, i.e., the JP conditions implemented higher illuminance levels by 200 lux and higher CCT by 500–1000 K than the US-specific conditions. Previous research demonstrates that bright light is an effective treatment for seasonal mood disorders as it helps improve affect [94], reduce distress [95], and improve mood in office workers [96]. However, the current study did not allow us to systematically isolate the impact of illuminance vs. CCT and, as such, further research is needed.

No significant differences were observed in metrics of mental stress, productivity, visual comfort, or perceived naturalness, although the measurement methodologies are all well adopted in the field and other previous studies on static lighting have shown promising results related to illuminance and CCT. Note that we used the findings of some outcomes to corroborate or explain the findings of the other outcomes. For example, the mood analysis showed that there were no significant differences in NA between static and dynamic lighting in either of the country-specific parameters. This may explain why the stress levels did not differ significantly across the conditions, according to the previous studies that explored the correlation between anxiety symptoms and ratings of NA [97].

### 5.2. Nighttime Impacts

Different from the daytime impacts, the nighttime impacts presented by dynamic lighting are mainly negative. For the JP-specific lighting conditions, participants’ daily sleep diary responses indicated a significant decrease in perceived sleep quality in the JP-D condition compared to the JP-T condition. This finding was further corroborated by the analysis of the measurement by the real-time sleep-tracking system on deep sleep, a restorative sleep stage. It was observed that the time spent in deep sleep progressively decreased throughout the JP-D condition, such that participants received approximately 24 min less deep sleep than in the JP-T condition. This finding indicates that although participants slept the same amount in both conditions, their sleep may not be as restful and therefore can lead to a significant decrease in their perceived sleep quality. 

For the US-specific lighting conditions, participants experienced approximately 32 min less in total sleep time. Despite this reduction in total sleep time, participants did not experience a corresponding drop in sleep quality, which means that even though participants slept less, they may have still gotten good quality sleep.

Participants had 20 min less light sleep in the US-D condition compared to the US-T lighting. This decrease corresponds with the decrease observed in total sleep time, as the two measures are linked due to light sleep accounting for approximately 50% of total sleep across the night in healthy people. In addition, a significant difference was observed in sleep onset, i.e., participants fell asleep approximately 20 min later after being exposed to US-D lighting. Despite the reduction in sleep time as well as the increase in sleep onset time, participants did not report experiencing significantly worse sleep quality. This means that although they went to sleep later and slept less overall, they may have still gotten quality sleep due to the fact that the amount of time spent in deep sleep and REM sleep did not change significantly.

### 5.3. Recommendations for Future Studies

One likely reason of the negative sleep impacts is that the dynamic lighting control profile may need to be further optimized to generate stronger effects beyond vision. In this study, the SPD peak value in the blue range in both dynamic and static lighting conditions was at approximately 450 nm. This is within the bioactive blue range (430–500 nm) [85]. However, it is not the most sensitive peak according to some other studies. For example, some scholars believe that the circadian stimulus peaks at approximately 460 nm [86] and the melanopsin SPD sensitivity peaks at approximately 480 nm [87]. A very recent study by Martin Moore-Ede et al. derived a novel human circadian potency spectral sensitivity curve which peaks at 477 nm [88]. Therefore, a lighting system that can generate the peak spectral power at approximately 460–480 nm may present potential stronger physiological, biological and behavioral effects. This can be a critical improvement in future studies.

Another explanation of the negative sleep impacts could be that participants’ exposure to bright-blue light in the morning was not sufficient to entrain their circadian rhythm. In other words, the difference in the total amount of blue light dosage between dynamic and static lighting conditions was not significant enough. Previous studies on static lighting that observed perceived sleep quality improvement had much longer exposures to blue lights and drastic CCT outcomes. For instance, Viola et al. exposed people to a static bright blue-enriched light of 17000 K for 8 h [30]. Therefore, future studies can consider revising the dynamic lighting profiles by increasing the morning illuminance/CCT levels and/or postponing the decrease in the illuminance/CCT levels in the afternoon.

The authors believe that further studies on the impact of dynamic lighting on human psychology and physiology are needed. Particularly, identifying the optimized levels of illuminance, CCT, and peak blue light spectrum in the morning versus in the afternoon would be the key for entrain human circadian systems. In future studies, a longer test period is recommended in which more lighting conditions can be introduced to unambiguously separate the contributions of illuminance and CCT. 

Another limitation of this study is that there were no measurements of the lighting exposure of subjects after office hours. We did not find any mature tools that can perform continuous eye-level lighting monitoring, but it may be available soon with the rapid development of sensor and wearable technologies. More comprehensive and objective monitoring of the participants’ behavior and sleep habits after work hours would also be beneficial. Such after-office-hour information may provide additional insights into the effectiveness of introducing dynamic lighting in offices and homes.

## 6. Conclusions

This study was designed to better understand the impact of dynamic LED lighting, with daily variation in illuminance and CCT levels, on the well-being and experience of the occupants in an office environment. Given that there are limited studies in the field for dynamic lighting, we designed this study to be broad and exploratory to cover more potential effects on human psychology and physiology. 

Fifteen participants were recruited to work in three office modules in a living lab for four months. Four lighting conditions were designed and implemented, including two static lighting conditions and two dynamic lighting conditions with a specific predefined control scheme. A prototype lighting system with enhanced illuminance and CCT control capabilities was installed, configured and programmed. The measured lighting data showed that the integrated operation of this lighting system and advanced shading technologies can successfully create a spatially uniform lighting environment and ensure the desired dynamic lighting profile. The lighting system design and control method can be a good reference for future human studies with indoor lighting.

The results showed that the impacts of dynamic lighting on daytime behavior were either positive or mixed. Specifically, a significant alertness increase was observed in the afternoon, indicating a potential solution to reduce the natural feelings of sleepiness during the workday. There was also a marginal benefit for mood. The nighttime impacts included a significant decrease in perceived sleep quality and sleep time after subjects were exposed to dynamic lighting. There was no significant difference observed for mental stress and productivity, our primary outcomes, indicating that the investigated dynamic lighting profiles would not be an effective approach to reduce occupant stress levels or increase occupant productivity. No significant differences were observed for visual comfort or perceived naturalness. The findings present additional insights into the non-visual impacts of dynamic lighting and give recommendations for further investigations.

## Figures and Tables

**Table 1:** Demographics breakdown of participants in this study.

**Race**	**Number**
White	12
Hispanic or Latino	2
Asian	1
**Income**	**Number**
Less than $10,000	1
$35,000 to less than $50,000	1
$50,000 to less than $75,000	4
$75,000 or more	7
Preferred not to answer	2
**Education**	**Number**
Some college or technical school	4
College graduate	11

**Table 2:** Chronotype information of participants in this study.

**Chronotype (Morning–Eveningness Questionnaire)**	**Number**
Definite morning	1
Moderate morning	5
Intermediate	8
Moderate evening	1
Definite evening	0
**Baseline Sleep and Stress Measures**	**Average (SD)**
Sleep Quality (Pittsburgh Sleep Quality Index)	5.33 (2.69)
Perceived Stress Scale	25.73 (6.94)
Job Stress Scale	12.13 (3.78)

**Table 3:**
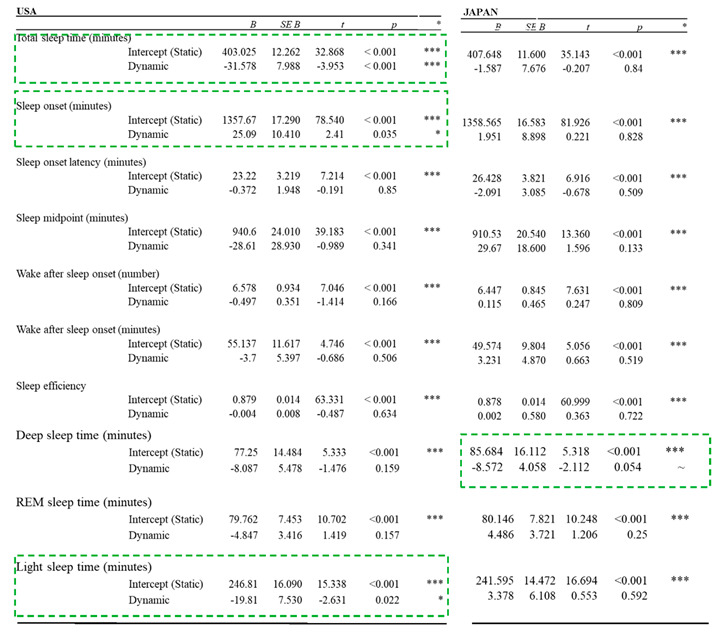
Results for continuous objective sleep measurements.

Note: Green dotted box for items with significant difference; *** (*p* < 0.001); ** (*p* < 0.005); * (*p* < 0.05); ~ (*p* < 0.1).

**Table 4:**
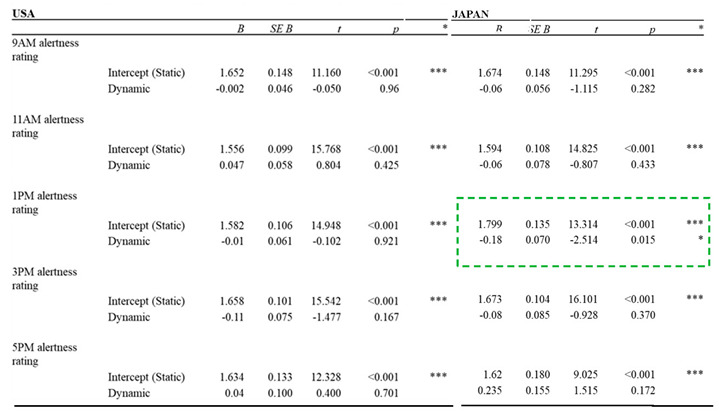
Results for daily alertness measurement.

Note: Green dotted box for items with significant difference; *** (*p* < 0.001); ** (*p* < 0.005); * (*p* < 0.05); ~ (*p* < 0.1).

**Table 5:**
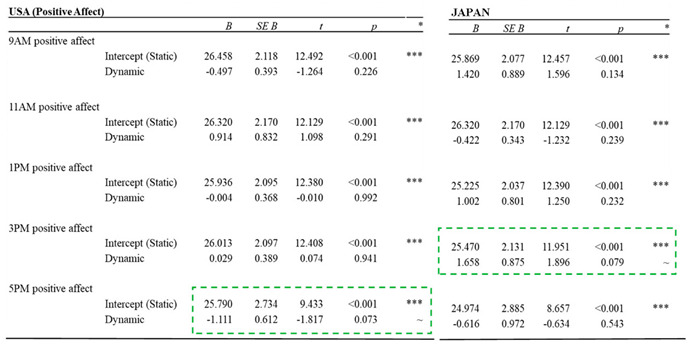
Results for the PANAS mood measurements (PA).

Note: Green dotted box for items with significant difference; *** (*p* < 0.001); ** (*p* < 0.005); * (*p* < 0.05); ~ (*p* < 0.1).
